# Aligning preclinical AML models with immunotherapy development: principles for model selection

**DOI:** 10.3389/fimmu.2026.1871786

**Published:** 2026-07-14

**Authors:** Efe Karaca, Pinar Ataca Atilla, Damian J. Green, Erden Atilla

**Affiliations:** 1 Division of Transplantation and Cellular Therapy Sylvester Comprehensive Cancer Center, Department of Medicine, Miller School of Medicine, University of Miami, Miami, FL, United States; 2 Ankara University Stem Cell Institute, Ankara University Graduate School of Health Sciences, Ankara, Türkiye; 3 GENYO, Centre for Genomics and Oncological Research: Pfizer, University of Granada, Granada, Spain

**Keywords:** acute myeloid leukemia, immunotherapy, *in vitro*, preclinical studies, *in vivo* models

## Abstract

Advances in immunotherapy for acute myeloid leukemia (AML) have revealed critical gaps in selection of appropriate preclinical models. Conventional cytotoxic and targeted therapies could be tested in relatively straightforward systems. Immunotherapies are inherently distinct from conventional therapies, as their activity is shaped by dynamic, context-dependent interactions between leukemic cells, immune effectors, and the bone marrow microenvironment that are difficult to replicate outside the patient. Failure to recapitulate these interactions may limit the clinical translatability of promising results. Recognizing these shortcomings, the field has moved through several generations of modeling platforms. Existing platforms capture distinct but incomplete aspects of AML biology: *in vitro* systems offer control but lack immune context, syngeneic models provide intact immunity but limited human relevance, patient-derived xenograft (PDX) models preserve patient biology but lack immunity, and humanized models partially integrate both but remain constrained. In this review, we trace the development of these systems and use that trajectory to build a practical framework for model selection in AML immunotherapy research. Translational relevance depends on selecting preclinical models that align with the specific therapeutic question, rather than relying on availability or perceived complexity. To operationalize this, we propose a mechanism-driven framework that maps therapeutic mechanisms to the most appropriate biological contexts across preclinical platforms. This framework progresses sequentially from controlled mechanistic studies to *in vivo* validation, guiding model selection at each stage. Instead of prioritizing any single system, it emphasizes a complementary, question-driven approach that leverages the distinct strengths of each model while accounting for their limitations.

## 1 Introduction

Acute myeloid leukemia (AML) is a heterogeneous hematologic malignancy defined by the clonal expansion of immature myeloid progenitors and disruption of normal hematopoiesis (1, 2). For decades, first line AML treatment has been anchored in intensive cytarabine- and anthracycline-based induction chemotherapy (3). Although the therapeutic landscape has broadened considerably with the introduction of targeted inhibitors, venetoclax-based regimens, and advances in transplantation, durable remissions remain difficult to achieve, and relapse continues to drive poor long-term outcomes, particularly among older and high-risk patients (4, 5).

A central factor underlying treatment failure and disease recurrence is the persistence of leukemia stem cells (LSCs). These cells possess self-renewal capacity, adapt to therapeutic selective pressures, and occupy protective bone marrow niches that confer resistance to both cytotoxic and targeted agents (6–8). The limitations of existing treatment strategies have generated growing interest in immunotherapeutic approaches, including monoclonal antibodies, bispecific T-cell engagers, chimeric antigen receptor (CAR)-based cellular therapies, and immune checkpoint inhibitors.

Immunotherapies, however, impose distinct demands on preclinical evaluation that conventional models were not designed to meet. Therapeutic efficacy and toxicity in this context are shaped by antigen density, effector-cell activation, cytokine dynamics, and immunosuppressive mechanisms within the bone marrow niche (9–11). Simplified *in vitro* platforms and early-generation murine models lacked the biological and microenvironmental complexity needed to capture these interactions (12–14), contributing to recurrent discordance between preclinical efficacy signals and clinical outcomes. In response, the preclinical toolkit has expanded to include primary patient-derived cultures, syngeneic murine leukemias, patient-derived xenografts (PDX), and increasingly sophisticated humanized platforms. While this expansion has enriched the available modeling landscape, it has also introduced a practical challenge: determining which system is best suited for a given immunotherapeutic objective. Existing reviews have provided valuable summaries of AML immunotherapy strategies, antigenic targets, clinical development, and animal model systems (15–17). However, these reviews generally address the therapeutic landscape and the preclinical models as separate domains. In contrast, the central premise of this review is that model selection should be driven by the biological requirements of the immunotherapeutic mechanism being investigated. Rather than cataloguing available platforms, we propose a mechanism-driven framework that aligns specific research questions—including antigen validation, immune-effector function, antibody-dependent activity, leukemia stem cell targeting, microenvironmental regulation, and immune-mediated toxicity—with the biological contexts required to answer them. By linking therapeutic mechanism to model selection across sequential stages of preclinical development, this framework provides practical guidance for investigators seeking to improve the translational relevance of AML immunotherapy studies.Relevant literature was identified through targeted searches of PubMed, Google Scholar, and reference lists of key articles using combinations of terms related to acute myeloid leukemia, preclinical models, immunotherapy, patient-derived xenografts, humanized mouse models, syngeneic models, organoids, three-dimensional culture systems, CAR-T cells, CAR-NK cells, bispecific antibodies, antibody-drug conjugates, and immune checkpoint blockade and some more. We prioritized peer-reviewed original studies and reviews that were directly relevant to AML model selection, immunotherapy mechanism of action, and translational interpretation. Recent publications emphasized where available, while landmark studies were included when they established foundational model systems, therapeutic concepts, or biological principles. The literature was curated by the authors to support a mechanism-driven framework for model selection rather than to provide an exhaustive systematic catalogue of all available studies.

## 2 *In vitro* modeling of AML: incorporating immune and microenvironmental features

*In vitro* AML models represent the earliest experimental platforms used to study leukemogenesis, therapeutic responses, and immune interactions, and they remain a foundational component of preclinical AML research (18, 19). Over the past several decades, these systems have evolved from simple immortalized leukemia cell lines to patient-derived cultures and increasingly complex platforms that incorporate elements of the bone marrow microenvironment and immune context (19–21). This progression reflects efforts to overcome earlier limitations and improve biological relevance, while maintaining mechanistic, hypothesis-driven investigation alongside *in vivo* validation.

### 2.1 Early *in vitro* systems: immortalized AML cell lines

Immortalized leukemia cell lines, including HL-60, THP-1, KG-1, MOLM-13, and MV4-11, laid much of the groundwork for early *in vitro* AML research (19). These lines provided controlled and reproducible platforms for studying leukemic proliferation, differentiation, apoptosis, and oncogenic signaling. Their rapid growth and ease of culture also made them well suited for genetic manipulation, including CRISPR/Cas9, shRNA, and lentiviral-based approaches (22). For many years, cell line models were central to mechanistic studies and preclinical therapeutic screening.

Despite their widespread use, immortalized AML cell lines exhibit limited biological fidelity. Continuous passaging promotes clonal drift and progressive loss of stem-like characteristics, while long-term *in vitro* adaptation introduces metabolic and phenotypic features that diverge from primary AML biology (19, 23). Recognition of clonal heterogeneity and leukemia stem cells (LSCs) in disease progression and resistance exposed the limitations of homogeneous cell line models.

### 2.2 Emergence of patient-derived systems: primary AML blasts

Primary AML blasts offer a patient-specific platform for evaluating antigen density, therapeutic vulnerabilities, and leukemia stem cell-associated features in a context that closely reflects clinical disease (1). Short-term *ex vivo* cultures enable mechanistic investigation of drug response, signaling pathway dependence, and stemness-related properties while preserving the biological heterogeneity of the original leukemia (20, 24). These systems are particularly valuable for validating findings obtained in immortalized cell lines and for capturing inter-patient variability — a critical consideration in immunotherapy research, where differences in antigen density and expression patterns can directly influence therapeutic efficacy.

Despite these strengths, primary AML blasts depend on supportive signals from the bone marrow microenvironment, including stromal contact and cytokine signaling, to maintain survival and stem-like properties. In the absence of these cues, primary blasts rapidly undergo apoptosis and lose functional stemness, limiting their utility for sustained culture and long-term functional analyses (25, 26).

### 2.3 From stromal support to immune co-cultures: evolving *in vitro* models of AML

Stromal co-culture systems have been developed to more accurately recapitulate the bone marrow niche, incorporating mesenchymal stromal cells, endothelial cells, and osteoblastic components (27). These platforms enhance leukemic blast survival, preserve leukemia stem cell-associated properties, and offer a controlled setting for investigating how microenvironmental interactions shape drug response and therapeutic resistance (28). By introducing niche-relevant cellular elements, stromal co-cultures improve biological fidelity, although they do not fully replicate the complexity of the intact bone marrow environment.

Beyond stromal support, the AML bone marrow microenvironment is shaped by hypoxic gradients, vascular niches, metabolic regulation, and inflammatory cytokine networks that collectively influence leukemia progression and therapeutic response. Hypoxia promotes leukemia stem cell maintenance and treatment resistance through activation of hypoxia-inducible factor (HIF)-dependent pathways and metabolic reprogramming (29, 30). Likewise, vascular niches regulate leukemic cell trafficking, survival, and interactions with immune effectors, while inflammatory mediators including IL-6, TNF-α, and TGF-β contribute to immune suppression and disease persistence (31, 32). Metabolic competition within the marrow microenvironment further shapes both leukemic and immune cell fitness through alterations in oxidative phosphorylation, amino acid utilization, and mitochondrial function (33, 34). These factors are increasingly recognized as critical determinants of immunotherapy efficacy but remain incompletely represented in many currently available preclinical platforms.

Researchers have made further efforts to narrow this gap, leading to the development of three-dimensional (3D) culture systems, including scaffolds, spheroid cultures, and hydrogel-based matrices designed to better replicate spatial architecture and mechanical properties of the bone marrow niche. Compared with traditional two-dimensional cultures, these 3D platforms provide a more physiological environment by enhancing cell-to-cell interactions. However, even the most advanced 3D systems lack full cellular diversity, functional vascularization, and the dynamic intercellular signaling characteristic of the *in vivo* microenvironment (35, 36).

As immunotherapeutic strategies gained traction in AML, a need emerged for *in vitro* systems that could actually model immune-mediated killing. This led to immune co-culture platforms, where AML cells are grown together with T cells, natural killer (NK) cells, CAR-engineered effectors, or immune cells redirected by bispecific antibodies (37, 38). The idea behind these systems is straightforward: placing leukemia and immune components in the same dish enables direct measurement of their interactions. In practice, they allow direct readout of effector cytotoxicity, antigen recognition thresholds, and cytokine release kinetics under controlled conditions (38). For early-stage work on CAR-based therapies and bispecific antibodies, co-cultures remain one of the more practical entry points before committing to costly *in vivo* experiments.

That said, what co-culture systems gain in control, they lose in complexity. They do not capture immune cell trafficking, spatial organization within the bone marrow niche, or crosstalk among diverse immune subsets, and systemic toxicities or immune amplification effects remain beyond the reach of *in vitro* approaches. This limitation extends across *in vitro* AML models more broadly.

### 2.4 Emerging organoid and microphysiological AML models

A newer generation of three-dimensional *ex vivo* platforms has emerged to bridge the gap between conventional co-culture systems and *in vivo* models. These include patient-derived organoids, induced pluripotent stem cell (iPSC)-derived marrow organoids, three-dimensional bioprinted bone marrow constructs, and bone marrow microphysiological systems (BM-MPS). These platforms aim to recreate structural, cellular, and biochemical features of the leukemic bone marrow niche while retaining greater experimental control than animal models. As highlighted by Silvestri and Chatterjee, these systems occupy an intermediate position between reductionist two-dimensional cultures and *in vivo* xenografts, offering a human-relevant level of complexity that may support studies of leukemia stem cell maintenance, drug resistance, immune interactions, and patient-specific therapeutic responses (39).

Unlike traditional stromal co-cultures, advanced three-dimensional platforms can incorporate mesenchymal stromal cells, endothelial elements, hematopoietic cells, extracellular matrix components, cytokine gradients, and, in some systems, immune populations within spatially organized architectures. Khan et al. developed human bone marrow organoids that support primary myeloid and lymphoid malignancy samples and allow therapeutic target validation in a microenvironmental context (40). Similarly, Cheung et al. demonstrated that engineered three-dimensional bone marrow niches can differentially modulate AML cell survival, phenotype, and drug response, supporting their potential role in personalized drug screening (41). Bioprinted AML models, such as those described by Alhattab et al., further add reproducible spatial control over cellular organization by incorporating leukemia cells together with stromal and endothelial components (42). Microfluidic leukemia-on-a-chip systems also provide opportunities to dissect niche-mediated resistance mechanisms in real time using patient-derived material, as shown by Ma et al. in leukemia bone marrow niche modeling (43).

These advantages are particularly relevant for AML, where niche-dependent survival, stemness, and therapy resistance are central barriers to durable disease control. Three-dimensional bioprinting and organ-on-chip technologies can enable controlled modeling of vascular gradients, oxygen tension, extracellular matrix composition, cytokine signaling, and stromal-leukemia interactions, thereby capturing aspects of AML biology that are difficult to reproduce in conventional suspension or two-dimensional cultures. BM-MPS platforms may also be useful for evaluating hematopoietic toxicity and biologic safety, especially when species-specific differences limit the predictive value of conventional animal models (44).

However, important limitations remain. Vascularization is still rudimentary in many systems, limiting perfusion, organoid size, and long-term culture stability. Functional immune and stromal compartments are also difficult to sustain over time, and incorporated immune cells may lose viability or functional diversity. This limitation is especially important for immunotherapy studies, where evaluation of antibody-, NK-, or T cell–based therapies require durable and functional immune-effector compartments. Organoid systems hold promise for immunotherapy assessment, but challenges related to immune-cell diversity, reproducibility, long-term culture stability, and clinical validation remain unresolved. Standardization, scalability, and cross-laboratory reproducibility are also major barriers (45).

Consequently, these platforms should currently be viewed as complementary rather than replacement models for PDX or humanized mouse systems. Their greatest near-term value may lie in advanced *ex vivo* screening, evaluation of niche-mediated resistance, leukemia stem cell biology, hematopoietic toxicity assessment, and patient-specific therapeutic prioritization. Within a model-selection framework, organoids, bioprinted marrow constructs, and BM-MPS platforms function best as an intermediate validation tier between reductionist *in vitro* assays and *in vivo* studies, rather than as definitive substitutes for immune-competent or humanized models required for full immunotherapy evaluation.

Cell lines, primary blast cultures, stromal systems, 3D platforms, and immune co-cultures each provide reproducible answers to defined questions, but none can recapitulate the integrated interactions between leukemia, immune effectors, and the microenvironment that shape disease *in vivo*. For immunotherapy research, transitioning to *in vivo* models is therefore not optional, but ultimately necessary. Before examining these *in vivo* platforms in detail, **Table 1** provides an overview of the full preclinical modeling landscape for AML, spanning both *in vitro* and *in vivo* systems. This comparison highlights how each platform differs in leukemia source, immune system status, experimental strengths, and translational applications, and serves as a reference framework for the sections that follow.

**Table 1:** Overview of preclinical AML model platforms and their key characteristics.

Model platform	Leukemia source	Immune system status	Major advantages	Major limitations	Primary translational applications
AML cell lines (*in vitro*)	Human (immortalized)	Absent	Highly reproducible, cost-effective, and amenable to genetic manipulation	Lack genetic heterogeneity and LSC fidelity; no microenvironmental or immune context	Pathway interrogation; high-throughput drug screening
Primary AML blasts (ex vivo)	Human (patient-derived)	Absent	Preserve patient-specific genetics and antigen expression	Limited viability; rapid loss of stemness without stromal support; no immune context	Target validation; assessment of inter-patient variability
Stromal and 3D *in vitro* systems	Human AML with stromal components	Absent	Model niche-dependent survival and therapeutic resistance; partially reconstruct the bone marrow microenvironment	Incomplete representation of *in vivo* tissue complexity	Microenvironment-mediated resistance studies; LSC survival modeling; toxicity screening
Immune co-culture systems	Human AML with immune effectors	Artificial/limited	Direct assessment of immune-mediated cytotoxicity and immune activation	No immune trafficking, persistence, or systemic toxicity evaluation	Early immunotherapy screening; mechanistic studies
Syngeneic mouse AML models	Murine AML	Fully immunocompetent	Intact immune regulation; genetically tractable; scalable	Murine leukemia biology and antigen expression may not fully reflect human AML	Mechanistic immunology; immune modulation studies
AML patient-derived xenograft (PDX) models	Human AML (patient-derived)	Absent	Preserve clonal heterogeneity, LSC hierarchy, and human antigen expression	Lack functional immune system; engraftment bias toward aggressive clones	Targeted therapy testing; resistance modeling
Humanized AML models	Human AML with human immune cells	Partial human immunity	Enable evaluation of human immune responses and immunotherapies	Incomplete immune reconstitution; high complexity and cost	CAR-T, CAR-NK, and bispecific antibody studies; immune-toxicity assessment

AML, acute myeloid leukemia; LSC, leukemic stem cell; PDX, patient-derived xenograft; CAR, chimeric antigen receptor; NK, natural killer.

## 3 *In vivo* AML modeling platforms

As outlined in **Table 1**, the *in vitro* systems discussed above, and the *in vivo* platforms described below differ fundamentally in their ability to capture systemic interactions among leukemia, host immunity, and the bone marrow niche. *In vivo* platforms allow researchers to study LSC behavior, microenvironmental dependence, immune modulation, and drug response under physiological conditions, which is precisely why they became indispensable for translational work and immunotherapy development (13).

### 3.1 Syngeneic mouse models (murine AML)

Syngeneic AML models have come a long way, from early transplantable leukemia lines to genetically defined systems built from engineered hematopoietic stem and progenitor cells. The first widely adopted models were C1498 myelomonocytic leukemia in C57BL/6 mice and the WEHI-3 model in BALB/c mice. C1498 transplantation allowed reproducible modeling of systemic leukemia progression and provided early insight into immune-mediated control of leukemic cells. WEHI-3, on the other hand, was primarily used for evaluating chemotherapy responses and splenic leukemic infiltration (46, 47). While these models offered practical advantages such as rapid engraftment and reproducible disease kinetics, they lacked defined genetic drivers and exhibited limited resemblance to human AML biology.

Later studies developed AML models by introducing leukemogenic fusion oncogenes into hematopoietic stem and progenitor cells (HSPCs) using retroviral vectors. This strategy enabled the generation of murine leukemias driven by fusion proteins such as MLL-AF9 and NUP98-HOXA9, which more closely recapitulate transcriptional aspects, differentiation arrest, and leukemia stem cell behaviors observed in human AML (48, 49). These HSPC-derived systems support the study of disease initiation, clonal evolution, and niche-dependent LSC maintenance within a controlled syngeneic context.

Further refinement of syngeneic AML modeling has been achieved through mutation-specific systems incorporating genetic lesions commonly observed in patients, including FLT3-ITD, NRAS, DNMT3A, TET2, and combinations of cooperating mutations (50–52). Using these models, researchers can examine how individual mutations and cooperating genetic events influence leukemogenesis, therapeutic response, and immune interactions in an immunocompetent host. By modeling patient-relevant mutational patterns, mutation-based syngeneic systems increase the biological relevance of murine AML studies.

Perhaps the most important feature of syngeneic AML models is that they preserve a fully intact immune system. This makes it possible to directly evaluate T-cell activation, NK-cell cytotoxicity, macrophage polarization, and cytokine signaling networks as leukemia progresses (53–55). For mechanistic studies of innate and adaptive immunity, immune evasion, and immunomodulatory interventions like checkpoint blockade or innate immune agonists, these models are hard to beat. They are also highly reproducible, cost-effective, and scalable for large cohorts — practical advantages that matter when designing studies with enough statistical power. The genetic tractability of these systems, whether through retroviral transduction or CRISPR-based engineering, adds another layer of experimental flexibility.

Syngeneic models rely on murine leukemia cells, they cannot fully capture the genetic diversity, antigen expression patterns, or epigenetic landscape of human AML. Clinically relevant targets, CD33, CD123, CLL-1, FLT3 differ substantially between mouse and human, which makes these platforms a poor fit for evaluating antigen-directed therapies such as CAR-T cells, CAR-NK cells, and bispecific antibodies (13, 56–59). Many of these models also run an aggressive disease course with limited LSC heterogeneity, so the slower, stem cell-driven progression that defines much of human AML is not well represented. Microenvironmental and stromal features in the murine bone marrow further diverge from their human counterparts, adding another source of translational uncertainty. Taken together, these gaps created a clear demand for *in vivo* platforms that could retain patient-specific leukemia biology and authentic human antigen expression, a need that ultimately drove the development and broad adoption of patient-derived xenograft systems.

### 3.2 Patient-derived xenograft models in AML

Patient-derived xenograft (PDX) models represent a major advance in AML modeling by enabling direct engraftment of primary human leukemia cells into immunodeficient mouse hosts, thereby preserving key biological features of patient disease that are absent in murine leukemia systems (60). AML PDX models are most commonly established in highly immunodeficient strains such as NOD/SCID/IL2Rγ^null (NSG) mice, which lack functional T cells, B cells, and natural killer (NK) cells, thereby enabling efficient engraftment of human hematologic malignancies (61).

A major advantage of AML PDX models is their ability to recapitulate patient-specific genetic alterations, clonal architecture, and the hierarchical organization of leukemia stem cells (LSCs). Importantly, AML PDX models preserve functional LSC populations and maintain hierarchical disease organization across serial transplantation. This feature distinguishes them from many rapidly proliferating cell line–based systems and makes them particularly valuable for evaluating therapies intended to eradicate disease-propagating stem cells and prevent relapse (6, 14). Studies have shown that dominant and subclonal populations are maintained across serial transplantation, supporting investigation of clonal evolution, therapy-driven selection, and mechanisms of relapse *in vivo* (14, 62). Therefore, AML PDX models are widely used for evaluating targeted therapies, combination regimens, and resistance mechanisms in a context that more closely reflects clinical disease than cell line–derived xenografts (63, 64). In this context, cell line–derived xenografts (CDX), established using AML cell lines in immunodeficient hosts, offer a reproducible and experimentally tractable system for early efficacy and mechanistic studies. However, their inability to capture genetic heterogeneity, leukemia stem cell hierarchy, and antigen variability inherent to primary AML limits their translational relevance, particularly in immunotherapy settings.

Another important advantage of AML PDX models is the preservation of authentic human antigen expression profiles, including clinically relevant targets such as CD33, CD123, CLL-1, and FLT3. This feature supports the evaluation of these antigens in preclinical immunotherapy studies and differentiates PDX models from murine leukemia systems lacking comparable antigen landscapes (56, 60, 65). This feature also makes AML PDX platforms particularly valuable for preclinical testing of antibody-based therapies, antibody–drug conjugates, and small-molecule inhibitors whose activity depends on human target expression (66). In addition, PDX models have been increasingly incorporated into functional precision-medicine strategies, where patient samples are screened against multiple therapeutic candidates to support biomarker development and treatment prioritization (13). The ability of PDX models to preserve subtype-specific genetic and phenotypic characteristics may be particularly important in AML, where molecularly defined disease subsets exhibit distinct stem cell programs, immune landscapes, and therapeutic vulnerabilities.

Despite the advantages, PDX models carry serious limitations when it comes to immunotherapy research. The deep immunodeficiency of NSG and related strains eliminates adaptive and innate immune responses entirely. Cellular immunotherapies like CAR-T or CAR-NK cells cannot be meaningfully evaluated in this context (67–69), and the dynamic immune–leukemia interactions central to immunotherapeutic efficacy and resistance are absent in standard PDX systems. Beyond the immune gap, additional limitations emerge. Engraftment efficiency is inconsistent across AML subtypes and tends to favor aggressive, highly proliferative clones, which means indolent disease populations and early leukemic states are likely underrepresented (61, 70). Another important limitation is that the engraftment process itself can introduce selection bias. AML subclones differ in their ability to survive transplantation, home to the murine bone marrow, and expand in the new host. As a result, aggressive or resistance-associated subclones may become preferentially enriched relative to the original patient sample, altering the apparent clonal architecture of the model (14, 61). This issue is particularly important for studies of relapse and therapeutic resistance, because clonal outgrowth after treatment may reflect both drug-induced selection and selection already introduced during xenotransplantation, rather than drug pressure alone. Therefore, resistance studies using AML PDX models should interpret post-treatment clonal changes in relation to both the original patient sample and the baseline engrafted leukemia.The microenvironment presents its own mismatch; human leukemia cells engraft within a murine bone marrow niche where stromal cells, cytokines, and extracellular matrix components are all mouse-derived (26, 71). Still, PDX models earn their place in the preclinical toolkit. They offer real gains in genetic, phenotypic, and antigenic fidelity over syngeneic systems. They enable *in vivo* pharmacodynamic assessment, support precision-medicine screening approaches, and remain among the best available platforms for studying targeted therapy responses and resistance mechanisms. The fundamental limitation in modeling the human immune system led the field to develop humanized AML models.

### 3.3 Alternative *in vivo* hosts: non-mammalian models of AML

Beyond mammalian systems, two non-murine *in vivo* platforms have gained traction in leukemia research: zebrafish (*Danio rerio*) and the chick embryo chorioallantoic membrane (CAM). Both share a defining feature; the host is immune-immature, with larval zebrafish lacking adaptive immunity and the early chick embryo lacking a developed lymphoid system, which allows human cells to engraft without immunosuppression (72, 73). What they offer in exchange is speed, low cost, scalability, minimal cell input, and real-time imaging in an intact organism, a combination mouse xenografts can barely match.

For AML, zebrafish xenografts are the more developed option, comparing the CAM. Human AML cell lines and primary blasts engraft and proliferate in larval hosts, where drug sensitivities can be read out within a 3 to 7 day window by fluorescence imaging or after dissociation, often alongside a simultaneous assessment of host toxicity (74). Transgenic zebrafish models complement these xenografts for longer-term study of leukemic driver genes (72). The CAM assay occupies similar ground, functioning as a rapid, vascularized, naturally immunodeficient host for engraftment and drug testing that has been proposed as a patient-derived xenograft alternative (73).

The same immune-immaturity that makes these hosts convenient is also what places them at the periphery of this framework. Neither sustains the functional human immune effectors that antibody-, NK-, or T cell–based therapies depend on, and the host’s own immunity is evolutionarily distant from human. These hosts have not gone untested for immunotherapy; CAR-T cells have been evaluated in both zebrafish and CAM xenografts (75, 76). Such efforts remain proof-of-concept demonstrations in limited, largely solid-tumor settings, however, rather than validated platforms for AML immunotherapy. Within the model-selection logic proposed here, zebrafish and CAM are therefore best understood as fast, accessible systems for engraftment, dissemination, drug screening, and toxicity triage that complement rather than replace the immune-competent murine models on which immunotherapy validation still depends. Their limitations, then, are not a reason to set these hosts aside but a reason to use them deliberately, for questions whose answers do not depend on the immune biology they lack.

### 3.4 Humanized AML models

Humanized AML models combine primary human AML cells with reconstituted human immune components in immunodeficient mice. In most protocols this means engrafting NSG or similar strains with human CD34^+^ hematopoietic stem and progenitor cells (HSPCs), then introducing patient-derived AML (77, 78). These models can support development of multiple human immune lineages: T cells, B cells, NK cells, and to varying degrees, myeloid populations. This provides direct evaluation of immune surveillance, immune evasion, cytokine signaling, and immune-mediated toxicity in the context of actual human leukemia (77, 79). Humanized platforms have become central to preclinical testing of CAR-T cells, CAR-NK cells, bispecific antibodies, checkpoint inhibitors, and macrophage- or NK-directed therapies (69, 80). For studying immune-mediated efficacy and toxicity, there is currently no true alternative that can recapitulate these processes in a physiological, *in vivo* context.

The platform has also been refined over time. Cytokine knock-in strains, expressing human IL-3, GM-CSF, SCF, M-CSF, or thrombopoietin, improve myeloid and NK-cell development and produce more robust leukemia–immune interactions (81–83). Introduction of human leukocyte antigen (HLA) transgenes is another advancement, enhancing antigen presentation and T-cell maturation to better model antigen-specific immune responses (84, 85). These engineering strategies have meaningfully improved the immune fidelity of humanized systems, though none fully resolve their underlying constraints.

Immune reconstitution varies considerably between animals, introducing heterogeneity that can complicate interpretation and limit reproducibility. T-cell lineages tend to dominate while myeloid compartments often remain underdeveloped (86, 87). The immune cells that do develop are typically derived from donor HSPCs that are not autologous to the engrafted leukemia, so patient-specific immune tolerance and antigen recognition are not faithfully modeled. Experimental windows are further narrowed by graft-versus-host disease, and the practical burden is high: these models are expensive, slow to generate, and show substantial animal-to-animal variability. On top of all this, the stromal, vascular, and cytokine environment surrounding both leukemia and immune cells remains murine, creating a hybrid niche whose influence on immune behavior and therapeutic response is difficult to quantify (26, 71, 88). Efficacy and toxicity readouts from humanized systems should therefore be treated as informative rather than predictive.

Humanized AML models represent the most advanced preclinical platforms currently available for immunotherapy development, uniquely enabling the study of human leukemia in the context of a functional human immune system. They bring together human leukemia biology and human immunity in a way that syngeneic and standard PDX models cannot. Yet this complexity is a double-edged sword: it enables greater biological relevance while requiring more rigorous experimental design and cautious interpretation. Importantly, this added sophistication does not obviate the need for simpler platforms—each addresses gaps that humanized models cannot fully capture.

Despite these advances, humanized AML models remain imperfect representations of human immunity. Immune reconstitution in humanized mice remains incomplete and variable, with persistent deficiencies in myeloid cell development, antigen presentation, and cytokine signaling (84). Similarly, Walsh et al. and Theocharides et al. emphasized that T-cell populations often predominate while myeloid and innate immune compartments remain incompletely developed, limiting faithful reproduction of human immune responses (86, 87). Furthermore, it was noted that cytokine networks are artificially reconstructed and frequently fail to recapitulate normal human hematopoiesis and immune homeostasis (77). An additional limitation is that immune cells are typically derived from donor hematopoietic stem cells that are not autologous to the engrafted leukemia, preventing accurate modeling of patient-specific immune tolerance and leukemia–immune co-evolution. HLA mismatch and xenogeneic influences may further affect antigen recognition and therapeutic responses (77, 84). Consequently, findings from humanized AML models should be interpreted as informative but not directly predictive of clinical efficacy or toxicity in patients.

**Figure 1:**
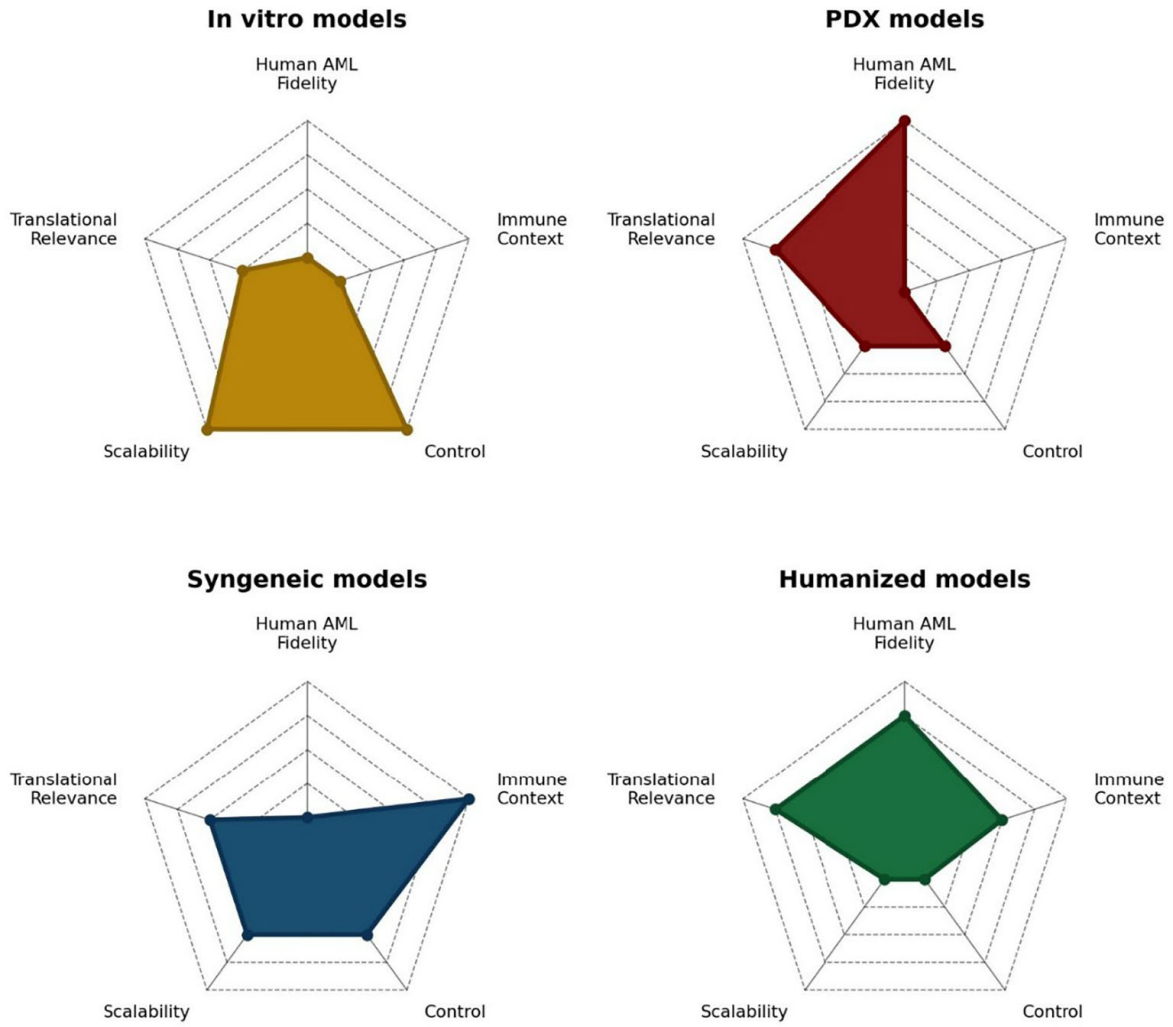
Illustrative comparison of preclinical AML model platforms across key dimensions relevant to immunotherapy development. The figure summarizes the relative strengths and limitations of major AML modeling systems, including *in vitro* cultures, advanced ex vivo platforms, syngeneic mouse models, patient-derived xenografts (PDX), and humanized mouse models. Dimensions shown include immune-system representation, translational relevance, scalability, control and experimental accessibility. The figure is intended as a conceptual framework to facilitate model selection.

As illustrated in **Figure 1**, each platform occupies a distinct niche across these evaluation criteria, and no single system dominates all dimensions. The practical implication is that model selection must be guided not by platform prestige or complexity, but by the specific immunotherapeutic question being asked.

## 4 Model selection strategies for immunotherapy development in AML

With the immunotherapy landscape in AML expanding rapidly, the question is no longer whether preclinical models are needed, but rather which models to use—and at what stage—to best address the underlying therapeutic question. The models reviewed above differ substantially in what they can represent: leukemia biology, immune context, systemic physiology. These are not interchangeable features, and choosing the wrong platform for a given question can undermine translational validity in ways that are difficult to recover from downstream (15, 77, 89).

The reality is that no single system captures everything. An additional consideration that cuts across all immunotherapeutic modalities is the ability of a model to preserve functional leukemia stem cell (LSC) populations. AML is hierarchically organized, with LSCs serving as the principal drivers of disease propagation, therapeutic resistance, and relapse (7, 8). Consequently, responses observed in bulk leukemic populations do not necessarily predict eradication of disease-propagating stem cells. As demonstrated by Shlush et al., relapse frequently originates from residual stem cell populations that survive initial therapy (14). Therefore, selection of preclinical models should consider not only therapeutic activity against bulk leukemia but also the ability of a platform to maintain LSC hierarchy, stemness, and niche-dependent interactions.

Each model illuminates certain aspects of AML biology while leaving others in the dark. What matters, therefore, is strategic alignment—rigorously matching the experimental model to the therapeutic modality and the specific biological question, rather than defaulting to systems based on familiarity or availability. In practice, this often means using multiple platforms in sequence or in parallel. To support these decisions, we propose a two-axis, therapy-aligned framework: one axis defines the therapeutic mechanism of action, the other the biological context the model must provide. This framework is applied across the major AML immunotherapy modalities in the sections that follow.

### 4.1 CAR-T antigen discovery and validation

The initial phase of CAR-T cell development centers on identifying target antigens that meet key criteria, including leukemia-restricted expression, adequate surface density, and stability across disease states*. In vitro* systems using AML cell lines and primary patient-derived blasts are well suited to this early phase, enabling controlled, high-throughput assessment of antigen expression across multiple samples and standardized comparison of CAR construct performance (80, 90, 91). These models are particularly valuable for excluding candidates with broad expression on normal hematopoietic progenitors and for evaluating combinatorial targeting strategies aimed at improving tumor selectivity and limiting off-tumor toxicity (92).

Despite their utility, *in vitro* systems do not reliably capture the antigen dynamics that emerge in the bone marrow microenvironment. AML patient-derived xenograft (PDX) models address this limitation by enabling validation of antigen retention within a more physiologically relevant niche and across leukemic subclones, a critical step for targets prone to downregulation or therapy-driven clonal selection (14, 61).

For advanced preclinical evaluation, humanized AML models currently represent the most informative *in vivo* platform, permitting simultaneous assessment of CAR-T expansion, persistence, and leukemic clearance in the presence of human immune components (69, 93, 94). However, inherent constraints of these systems, including incomplete immune reconstitution and potential allogeneic interactions, require that findings be interpreted with caution and validated across complementary model systems.

In addition to evaluating antigen expression on bulk AML blasts, antigen validation studies should consider expression patterns on leukemia stem cells, since persistence of antigen-positive LSC populations may contribute to relapse despite effective elimination of the bulk disease compartment. Models capable of preserving LSC biology may therefore provide additional translational value during target selection and validation.

### 4.2 Antibody-based therapies: antibody–drug conjugates and Fc-dependent mechanisms

Antibody-based agents occupy a distinct position in the AML immunotherapy landscape because their modeling requirements dictate a sharp divide based on mechanism of action. Antibody–drug conjugates (ADCs) kill primarily through antigen binding, internalization, and intracellular payload release, depending little on host immune effector function (95). In contrast, naked or Fc-engineered antibodies act through antibody-dependent cellular cytotoxicity (ADCC), antibody-dependent cellular phagocytosis (ADCP), complement activation, or macrophage checkpoints (e.g., CD47–SIRPα). These mechanisms require functional immune effectors and appropriate Fc–Fc receptor interactions (54, 96).

For ADCs, this relative immune independence simplifies model selection. *In vitro* assays using AML cell lines and primary blasts allow controlled assessment of antigen density, internalization, payload sensitivity, and bystander killing (56, 66, 95). However, because targets like CD33 and CD123 are also expressed on normal myeloid progenitors, colony-forming unit assays remain essential for gauging hematopoietic liability (54, 97). *In vivo*, the lack of reliance on a host immune system makes standard cell line–derived xenografts and PDX models highly informative for assessing efficacy, pharmacodynamics, and antigen-dependent killing (54, 66). This is exemplified by gemtuzumab ozogamicin, the first ADC approved for AML (98). Unlike cellular immunotherapies—which struggle in standard NSG-based PDX models due to the absence of an immune context (54, 56)—ADCs can be robustly evaluated in these conventional platforms.

Fc-dependent antibodies impose the opposite requirement. Because NK cells, macrophages, and complement are absent or functionally limited in standard PDX hosts, these models cannot reliably capture ADCC, ADCP, or complement-dependent cytotoxicity (98, 99). Consequently, early evaluation is better suited to *in vitro* effector–target co-cultures using human NK cells or monocyte-derived macrophages (54, 100, 101). Translating these findings *in vivo* remains challenging due to substantial differences between mouse and human Fc receptors, which can alter how human antibodies behave in murine systems (102). Humanized models with improved NK-cell and myeloid reconstitution—particularly FcγR-humanized strains—are more appropriate, though they remain constrained by variable and incomplete reconstitution (77, 82, 83, 102). CD47–SIRPα-directed therapies illustrate this well: their reliance on macrophage-mediated phagocytosis demands evaluation in macrophage-competent systems (101, 102).

Ultimately, antibody-based therapies reinforce this framework’s central principle: the model must follow the mechanism. Yet, even appropriately matched platforms retain characteristic limitations. *In vitro* systems lack systemic pharmacokinetics and toxicity profiles, standard xenografts rely on a murine niche, and humanized strains often exhibit variable, myeloid-poor reconstitution. Because no single system is comprehensive on its own, efficacy and safety are most reliably established by combining complementary models.

### 4.3 On-target/off-tumor toxicity

One of the harder problems in AML immunotherapy is that many candidate antigens are not truly leukemia-specific, they also are expressed on normal hematopoietic progenitors*. In vitro* co-culture assays using normal CD34^+^ progenitors or differentiated myeloid populations can flag overt cytotoxicity as a first pass, but they miss immune amplification effects, spatial restriction within tissues, and anything systemic (93, 97, 103). Colony-forming unit (CFU) assays remain the standard functional readout in this context, quantifying the impact of candidate therapies on myeloid, erythroid, and multipotent progenitor output and providing a more granular picture of hematopoietic toxicity than surface phenotyping alone (38, 104). More recently, bone marrow micro-physiological systems; microfluidic chips seeded with human CD34^+^ cells, stromal components, and optionally autologous T cells have shown early promise for profiling lineage-specific hematopoietic liabilities and immune-mediated on-target toxicity of biologics in a more physiologically integrated format (44).

Syngeneic models offer limited utility in this context. Murine antigen expression patterns differ sufficiently from humans, limiting the translatability of safety conclusions (105, 106). Humanized AML models get closer, they allow some evaluation of immune-mediated toxicity, cytokine release, and hematopoietic disruption in a living host (77, 107). But even these platforms fall short of reproducing clinical toxicity syndromes like cytokine release syndrome (CRS) or immune effector cell-associated neurotoxicity syndrome (ICANS) in any reliable way.

The clear conclusion is that antigen safety assessment in AML remains inherently imperfect; no preclinical platform can fully predict clinical outcomes in patients. Conservative interpretation, layered across multiple model systems, is not just good practice, it is the only responsible approach given current tools.

### 4.4 Myeloid compartment biology and innate immune regulation

Immune evasion in AML is largely driven by innate immune populations such as macrophages and myeloid-derived suppressor cells, which remodel the leukemic microenvironment in ways not fully accounted for by adaptive immunity. Understanding how these cells are polarized, how they suppress anti-leukemic responses, and how they might be therapeutically redirected requires models with a functional myeloid compartment.

*In vitro* co-culture systems offer an accessible entry point. When primary AML blasts are cultured with monocyte-derived macrophages or MDSC-enriched populations, they can drive polarization toward immunosuppressive phenotypes, including M2-like macrophage skewing and MDSC-mediated T cell suppression, in controlled settings that facilitate interrogation of cytokine pathways and cell-contact dependencies (108). More recently, co-culture of AML cells with M2-polarized macrophages has been shown to confer phagocytic resistance and enhance mitochondrial metabolism in leukemic blasts, offering mechanistic insight into how the innate compartment directly supports leukemia persistence (109). These platforms are valuable for hypothesis generation and pathway-level work, but they strip away the spatial and multi-cellular complexity of the bone marrow niche and cannot capture systemic feedback loops.

Syngeneic AML models are the natural starting point for *in vivo*. Their intact immune system supports mechanistic work on myeloid polarization, cytokine signaling, and immune suppression within the leukemic niche (104, 110). The limitation is that these systems are entirely murine—the leukemia, target antigens, and immune compartments—so their translational relevance is inherently constrained (49). Humanized models, particularly cytokine knock-in strains, attempt to address this gap by enabling *in vivo* development of human myeloid and NK cell populations (82, 99, 111). While this represents a meaningful advance, innate immune reconstitution remains variable, such that findings from any single cohort are best interpreted cautiously and validated across complementary platforms before drawing firm conclusions (77).

### 4.5 NK-cell–based immunotherapies

NK-cell–based therapies occupy a distinct immunological niche at the interface of innate and adaptive immunity, and this hybrid functional identity imposes specific requirements on preclinical modeling systems. Early-stage questions around NK-cell activation, target recognition, and antibody-dependent cellular cytotoxicity can be addressed reasonably well *in vitro* using cytotoxicity assays and immune co-culture systems (112, 113). These platforms cannot resolve whether NK cells will effectively traffic to relevant anatomical compartments, persist at levels sufficient for therapeutic activity, or maintain functional competence under the immunosuppressive pressures of the leukemic microenvironment. More specifically, standard co-culture systems often fail to capture how the leukemic microenvironment suppresses NK cells before they reach their targets. TGF-β plays a major role in this process. At levels commonly found in AML bone marrow, it gradually reduces NK cell cytotoxicity, lowers the expression of activating receptors such as NKG2D and CD16, and weakens effector function over time (114). To address this limitation, ex vivo systems that incorporate this suppressive pressure are increasingly used to evaluate rescue strategies, including TGF-β receptor blockade, before moving into *in vivo* studies. In parallel, cytokine-induced memory-like NK cells, generated through brief pre-activation with IL-12, IL-15, and IL-18, have introduced a distinct set of preclinical considerations (115). These cells display enhanced anti-leukemia activity and improved persistence compared to conventional NK-cell preparations, and have already shown clinical activity in relapsed AML (116). Modeling their behavior requires platforms that can capture not just acute cytotoxicity but durability and *in vivo* expansion over time.

For those questions, humanized AML models are currently the best available option. Strains engineered to express human cytokines that support NK-cell development and survival make these systems considerably more informative than standard humanized platforms (117, 118). Even so, NK-cell reconstitution varies enough between animals that results from a single experiment should be treated carefully. Replication across independent cohorts is not optional; it is essential for distinguishing preliminary observations from robust, reproducible findings.

### 4.6 Bispecific antibodies and T-cell–redirecting therapies

The fundamental mechanism of bispecific antibodies relies on the coordinated engagement of a human immune effector and a target antigen expressed on human leukemia cells. Both components must be present and functionally competent for activity to be observed, which substantially restricts the range of preclinical models capable of providing meaningful evaluation. *In vitro* immune co-cultures handle early work well: proof-of-concept testing, potency ranking, activation threshold characterization (119–121), Pharmacokinetics, the evolution of immune exhaustion over time, and systemic toxicity are inherently *in vivo* phenomena that cannot be meaningfully recapitulated *in vitro*.

Humanized AML models currently provide the most informative *in vivo* platform for evaluating bispecific candidates, as they enable concurrent engagement of human immune effectors and targeting human leukemia cells within the same host (77, 122). That said, the limitations discussed earlier do not disappear here. Immune reconstitution remains incomplete, cytokine signaling is frequently dysregulated, and a meaningful gap persists between responses observed in humanized mouse models and those seen in patients. Layering *in vitro* and *in vivo* data from multiple systems is less of a recommendation and more of a requirement if the goal is to move a bispecific program forward with any confidence.

### 4.7 Immune checkpoint blockade

Checkpoint inhibitors require a context that most AML models fail to recapitulate: a fully functional immune system in dynamic equilibrium with the tumor (123). In the absence of this baseline, there is no meaningful inhibitory axis to relieve. Syngeneic models come closest on the immune side; they preserve intact checkpoint biology and allow mechanistic studies of immune exhaustion, T-cell reinvigoration, and rational combination strategies in an immunocompetent host (124, 125). The limitation, as with all syngeneic systems, is that both the leukemia and its target antigens are murine. Consequently, the extent to which these findings translate to human biology remains inherently uncertain.

Humanized AML models incorporate human immune components into the system but encounter a different set of limitations. The immune reconstitution in these systems is often too incomplete or too skewed to generate the kind of mature, diverse immune landscape that durable checkpoint responses seem to require (16, 126). This leaves checkpoint blockade in a clear modeling gap: syngeneic systems provide intact immune circuitry but lack human disease context, whereas humanized models offer the correct species context but with incomplete and immature immunity. As a result, this remains a persistent translational bottleneck in AML immunotherapy, with no existing platform fully resolving the disconnect. In practical terms, syngeneic AML models currently represent the most appropriate platform for mechanistic studies of checkpoint blockade because they preserve intact immune circuitry and enable investigation of T-cell exhaustion, immune reinvigoration, and rational combination strategies *in vivo* (124, 125). However, investigators should recognize that both leukemia and immune compartments are murine, limiting direct extrapolation to human disease. Consequently, findings derived from syngeneic systems should be considered hypothesis-generating and ideally complemented by studies using primary patient samples, ex vivo immune co-culture systems, or humanized models before translational conclusions are drawn.

### 4.8 Leukemia stem cell–targeting strategies

The effects of a therapy on leukemic progenitor cells can be evaluated using *in vitro* assays such as colony-forming unit assays and long-term culture-initiating cell (LTC-IC) assays with stromal support (127). These assays measure self-renewal capacity and clonal output under controlled conditions. However, they remove leukemic stem cells from their native niche, which becomes a key limitation when studying therapies that rely on disrupting those microenvironmental interactions.

Therapies targeting LSCs require model systems in which these cells are preserved within a bona fide hierarchical organization—maintaining functional stem–progenitor relationships, niche dependence, and clonal architecture—rather than being represented as a homogeneous bulk population. PDX models are the strongest option here. They maintain stem cell populations and clonal architecture through serial transplantation, which makes them well suited for evaluating whether an LSC-directed therapy actually reaches and eliminates the cells that drive long-term disease maintenance (14, 62, 128). Syngeneic and other rapidly progressive murine models tend to be less informative for this purpose. Their aggressive kinetics favor fast-proliferating bulk populations and often underestimate the role of slow-cycling LSCs, precisely the cells responsible for persistence and eventual relapse.

### 4.9 Modeling cytokine release syndrome and neurotoxicity

Cytokine release syndrome (CRS) and immune effector cell associated neurotoxicity (ICANS) are among the most serious complications of immunotherapy, and they are also among the hardest to model. As an initial screening step, *in vitro* cytokine release assays using whole blood or PBMCs incubated with the therapeutic candidate can detect early inflammatory signals such as IL-6, TNF-α, and IFN-γ, and help rank candidates based on their potential to trigger systemic immune activation (129, 130). Importantly, these assays are useful for comparative candidate ranking and mechanistic assessment of inflammatory potential but should not be considered predictive models of patient-specific CRS or ICANS severity, as they fail to capture critical determinants such as tumor burden, immune reconstitution, endothelial activation, blood-brain barrier dysfunction, and multi-organ interactions that contribute to clinical toxicity (129–134). While these assays are useful for identifying relative safety signals and prioritizing therapeutic candidates, they do not reflect the complexity of the *in vivo* setting and therefore cannot reliably predict clinical toxicity outcomes. In particular, they lack critical determinants such as tumor burden, dynamic immune reconstitution, and multi-organ interactions—factors that play a central role in shaping the onset and severity of cytokine release syndrome in patients.

Humanized AML models can reproduce parts of the *in vivo* responses, including certain patterns of cytokine dysregulation and limited immune activation. However, they do not reliably capture the full clinical spectrum of these toxicities, particularly their multi-organ involvement and variable severity observed in patients (131–133). Neurotoxicity remains even more difficult to model. ICANS involves disruption of the blood–brain barrier, endothelial activation, and CNS inflammation driven largely by monocyte-derived cytokines. These processes depend on intact neurovascular architecture and species-specific immune–endothelial interactions that are not faithfully reproduced in current mouse models (134, 135). As a result, preclinical systems are best suited to provide mechanistic insight rather than clinical prediction. This distinction is important and should be clearly stated when preclinical toxicity data are used to guide dose selection or define safety margins.

**Figure 2:**
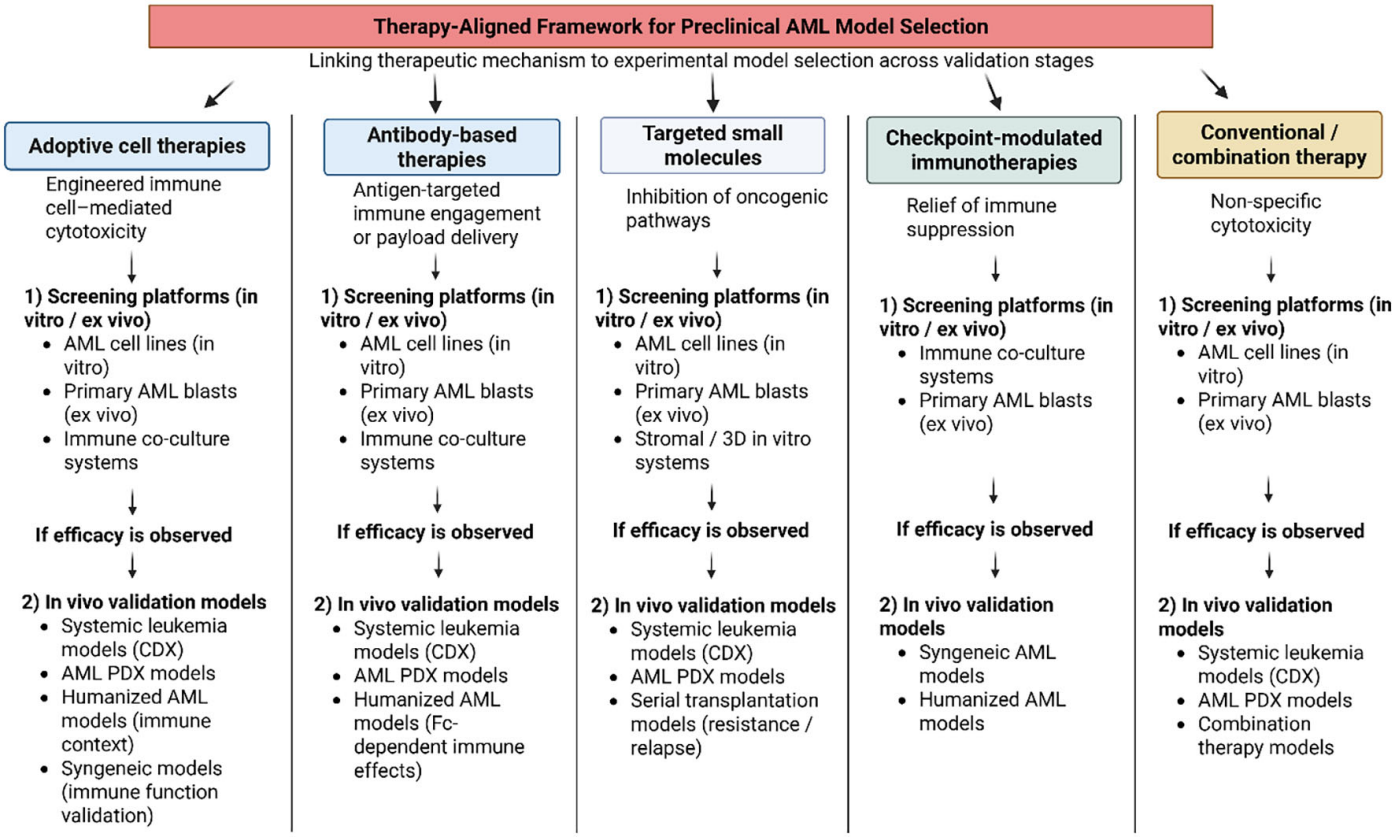
Therapy-aligned framework for preclinical AML model selection. Schematic overview linking five major therapeutic modalities to recommended preclinical model systems across two sequential validation stages. For each modality, the primary mechanism of action (listed below the category header) informs the selection of *in vitro* and ex vivo screening platforms (Stage 1). Progression to *in vivo* validation (Stage 2) is contingent on demonstration of efficacy in earlier-stage systems. Model recommendations reflect the principles discussed in Sections 2–4 and are intended to complement the question-specific guidance provided in **Figure 3**. AML, acute myeloid leukemia; CDX, cell line–derived xenograft; PDX, patient-derived xenograft; HMA, hypomethylating agent.

**Figure 3:**
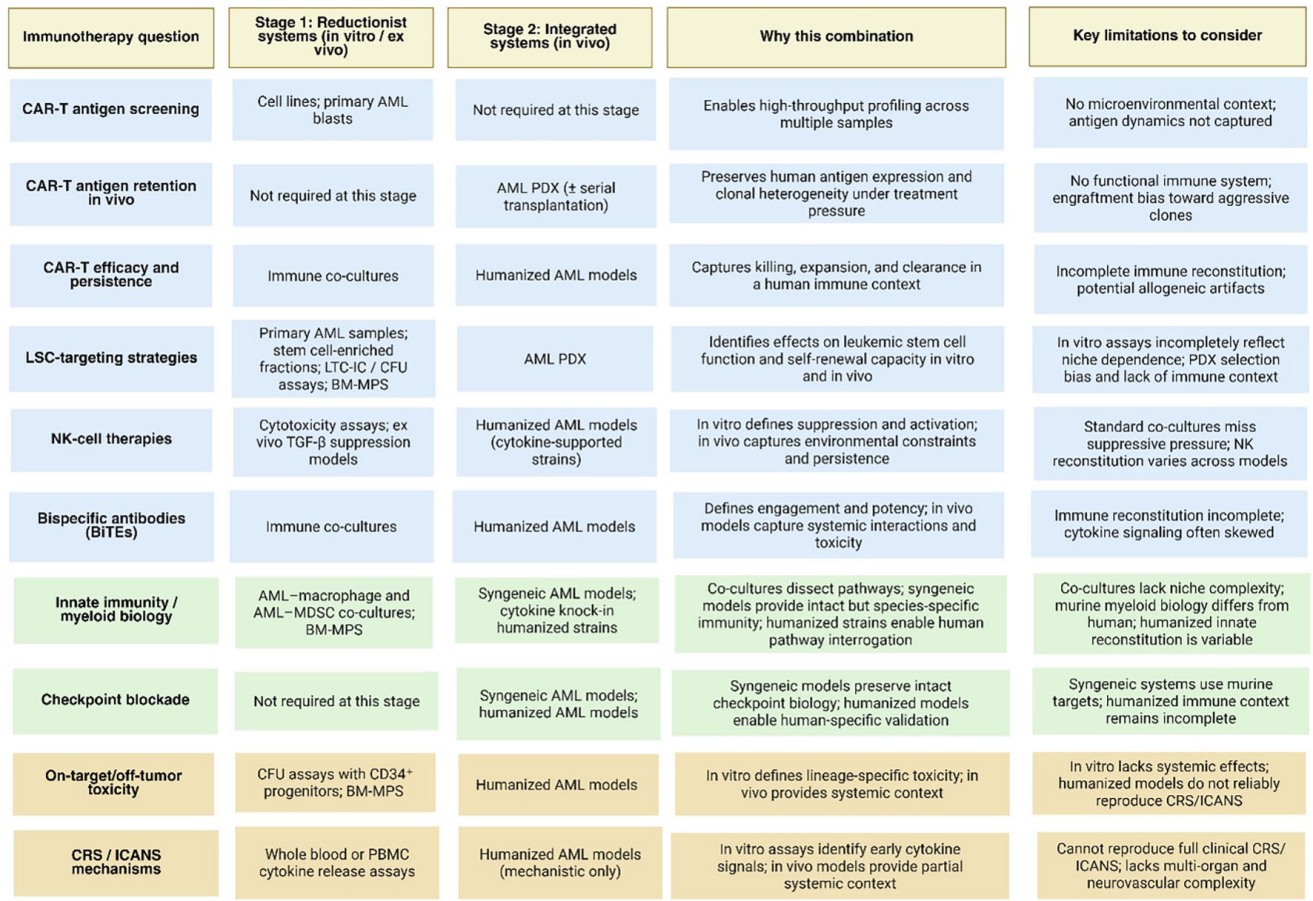
Model selection guide for AML immunotherapy development. Practical reference framework mapping ten key immunotherapy questions to recommended preclinical platforms across two sequential validation stages. Stage 1 (reductionist systems) encompasses *in vitro* and ex vivo platforms suited for early mechanistic screening, while Stage 2 (integrated systems) identifies *in vivo* models appropriate for physiological validation. For each question, the rationale for the recommended platform combination and its principal limitations are provided. Entries marked “Not required at this stage” indicate that the corresponding validation layer does not apply to that modality. Rows are color-coded by category: blue denotes effector-based therapies (CAR-T, bispecific antibodies, NK-cell therapies), green denotes immune biology and microenvironment-related questions (innate immunity/myeloid biology, checkpoint blockade, LSC-targeting strategies), and orange denotes toxicity assessment (on-target/off-tumor toxicity, CRS/ICANS mechanisms). This framework is intended to complement the modality-based logic presented in **Figure 2** and the detailed discussion in Sections 4.1–4.9. AML, acute myeloid leukemia; BiTEs, bispecific T-cell engagers; BM-MPS, bone marrow microphysiological system; CAR-T, chimeric antigen receptor T cell; CFU, colony-forming unit; CRS, cytokine release syndrome; ICANS, immune effector cell-associated neurotoxicity syndrome; LSC, leukemia stem cell; LTC-IC, long-term culture-initiating cell; MDSC, myeloid-derived suppressor cell; NK, natural killer; PBMC, peripheral blood mononuclear cell; PDX, patient-derived xenograft; TGF-β, transforming growth factor beta.

To help consolidate the reasoning outlined across these subsections, **Figure 2** provides a visual overview of how the mechanism of action of each therapy class determines which preclinical models are most appropriate at each validation stage. **Figure 3** expands on this logic in greater detail, mapping each immunotherapy question to the most appropriate preclinical platform across both reductionist and integrated validation stages, along with the rationale for each recommendation and its key limitations. Together, these two tools can be used to verify whether the selected platform provides the biological context demanded by the therapy’s mechanism and the validation objectives of the study.

Although the framework presented here identifies the biologically most informative models for specific immunotherapeutic questions, several practical considerations may influence model selection in real-world research settings. Not all AML samples are equally amenable to preclinical modeling; in particular, certain AML subtypes may exhibit poor or inconsistent engraftment in xenograft systems due to lower proliferative capacity, niche dependence, or limited leukemia stem cell frequency. As a result, some disease states may be underrepresented in PDX-based studies. In addition, advanced platforms such as humanized mouse models, organoid systems, and microphysiological bone marrow models require specialized expertise, infrastructure, and financial resources that may not be available to all investigators. Consequently, model selection should balance biological relevance with practical feasibility, and complementary use of alternative platforms may often represent the most realistic strategy for translational AML research.

## 5 Conclusion

Preclinical AML models have evolved significantly over the past few decades. Early approaches relied on immortalized cell lines and short-term blast cultures, which were useful for mechanistic studies but did not capture stem cell hierarchies, the bone marrow niche, or immune interactions. Today, the field includes a broader set of models such as syngeneic murine leukemias, patient-derived xenografts, and humanized mouse systems. Each generation of models emerged because the previous one left specific questions unanswered. That pattern of problem driven innovation is itself one of the clearest lessons from the field’s history.

With the expanding use of immunotherapy in AML, the selection of appropriate preclinical models has become increasingly critical. These therapies depend on interactions between leukemic cells, immune cells, and the bone marrow microenvironment, which are highly context dependent. No preclinical platform fully reflects this complexity. This pattern has been observed consistently, with many promising preclinical results not translating in clinical settings. Model sophistication, on its own, does not guarantee predictive accuracy. A complex model used for the wrong question can mislead just as effectively as a simple one. This underscores the need to treat model selection as an integral part of study design, since it directly determines how efficacy and safety data can be understood, rather than as a minor technical consideration. The framework we have outlined here is built around a straightforward principle: aligning the model to the treatment or the question. *In vitro* systems remain the right starting point for antigen discovery, mechanistic dissection, and early screening. Syngeneic models provide the immunocompetent setting needed for studying myeloid biology and immune regulation. PDX platforms preserve patient-specific genetics, clonal architecture, and LSC populations in ways that no murine leukemia system can. Humanized models currently come closest to enabling the study of human immune–leukemia interactions *in vivo*, though their constraints are well documented and should not be understated.

Going forward, progress will depend not just on building more complex systems, but on understanding what each platform can truly offer. Emerging patient-derived organoids, 3D bone marrow models, and organ-on-chip platforms may help bridge the gap between reductionist *in vitro* systems and animal models by incorporating more physiologic niche architecture, stromal interactions, and immune components. In parallel, spatial biology and single-cell multiomics are likely to improve how preclinical models are benchmarked against patient AML by resolving clonal architecture, immune composition, niche localization, and therapy-induced changes at higher resolution. Artificial intelligence-guided model selection may further support this process by integrating genomic, phenotypic, microenvironmental, and therapeutic-response data to identify which platform or combination of platforms best fits a given experimental question. However, increased model complexity does not inherently translate into greater clinical relevance. As immunotherapy strategies in AML continue to evolve, appropriate model selection and cross-validation across complementary platforms will remain critical determinants of experimental rigor and translational relevance. Future preclinical models will increasingly need to incorporate not only immune and stromal components but also hypoxic, metabolic, vascular, and inflammatory features of the bone marrow niche to more accurately predict immunotherapeutic responses.
